# Africa’s Nomadic Pastoralists and Their Animals Are an Invisible Frontier in Pandemic Surveillance

**DOI:** 10.4269/ajtmh.20-1004

**Published:** 2020-09-10

**Authors:** James M. Hassell, Dawn Zimmerman, Eric M. Fèvre, Jakob Zinsstag, Salome Bukachi, Michele Barry, Mathew Muturi, Bernard Bett, Nathaniel Jensen, Seid Ali, Stace Maples, Jonathan Rushton, Rea Tschopp, Yahya O. Madaine, Rahma A. Abtidon, Hannah Wild

**Affiliations:** 1 Global Health Program, Smithsonian Conservation Biology Institute, Washington, District of Columbia;; 2 Department of Epidemiology of Microbial Disease, Yale School of Public Health, New Haven, Connecticut;; 3 Institute of Infection, Veterinary and Ecological Sciences, University of Liverpool, Liverpool, United Kingdom;; 4 International Livestock Research Institute, Nairobi, Kenya;; 5 Swiss Tropical and Public Health Institute, Basel, Switzerland;; 6 University of Basel, Basel, Switzerland;; 7 Institute of Anthropology, Gender and African Studies, University of Nairobi, Nairobi, Kenya;; 8 School of Medicine, Stanford University, Stanford, California;; 9 Center for Innovation in Global Health, Stanford University, Stanford, California;; 10 Kenya Zoonotic Disease Unit, Ministry of Agriculture, Livestock and Fisheries, Nairobi, Kenya;; 11 Jigjiga University, Jigjiga, Ethiopia;; 12 Stanford Geospatial Center, Stanford University, Stanford, California;; 13 Armauer Hansen Research Institute, Addis Ababa, Ethiopia;; 14 Department of Surgery, University of Washington, Seattle, Washington

## Abstract

The effects of COVID-19 have gone undocumented in nomadic pastoralist communities across Africa, which are largely invisible to health surveillance systems despite the fact that they are of key significance in the setting of emerging infectious disease. We expose these landscapes as a “blind spot” in global health surveillance, elaborate on the ways in which current health surveillance infrastructure is ill-equipped to capture pastoralist populations and the animals with which they coexist, and highlight the consequential risks of inadequate surveillance among pastoralists and their livestock to global health. As a platform for further dialogue, we present concrete solutions to address this gap.

Conducting disease surveillance of human and animal populations in regions of high animal-to-human spillover risk, where a critical window of opportunity exists to prevent outbreaks from propagating, is of high global health and economic importance.^1^ One such area is Africa’s drylands, which constitute roughly 43% of the continent’s land area and are home to between 50 and 200 million nomadic pastoralists, who subsist on herds of livestock that move between water sources and seasonal grazing areas in biodiverse landscapes that are known to harbor emerging pathogens.^2^ Remoteness and the mobility necessitated by livestock management practices that frequently cross national borders have led to the inadequate integration of people and their animals that exist on the front line of spillover risk into health services.^3^

The COVID-19 pandemic has thrown this issue sharply into focus. The absence of peer-reviewed research, commentary, and reporting on the impacts of COVID-19 for pastoralists demonstrates that much-needed evidence is currently inaccessible to those tasked with monitoring the health of a substantial proportion of Africa’s people and animals. Of 187 reports published in 2020 on the epidemiology of COVID-19 in Africa indexed in PubMed, zero feature data from pastoralist communities or mention these groups in the context of national management plans for the virus. With sizeable populations unaccounted for in surveillance programs, regional response strategies may be compromised,^4^ and disease-related morbidity among these groups may go unrecognized, and therefore unmitigated, by governments. Given that large swathes of sub-Saharan Africa are considered hotspots for wildlife-borne emerging zoonotic diseases,^5,6^ pastoralists, whose lives are characterized by constant interactions with their livestock which come into repeated contact with wildlife, are at high risk from emerging infectious diseases (EIDs). COVID-19 should serve as a forewarning that inadequate surveillance in these environments limits governments’ ability to detect outbreaks of novel pathogens and respond appropriately before they spread further.

To improve representation of pastoralists and their animals in surveillance systems, we recommend a hub-and-spoke model^7^ to extend existing, centralized healthcare systems (“hubs”) into underserved, remote locations. This could be achieved by establishing a workforce of One Health extension personnel, equipped with portable diagnostics, that serve as “spokes,” with adequate provisions for populations whose movements regularly cross national borders (Figure 1).

**Figure 1.**
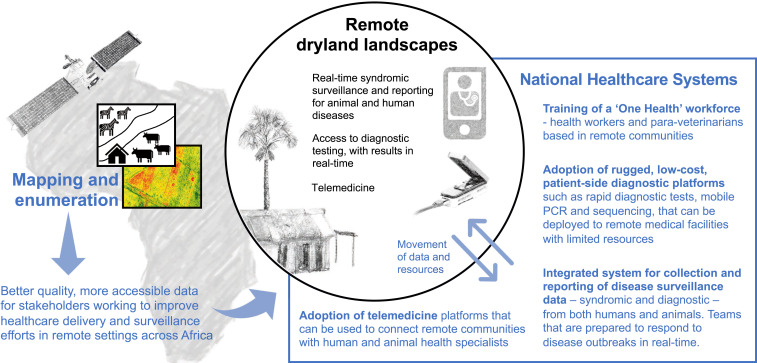
Steps that can be taken to enhance human and animal disease surveillance and healthcare services for nomadic populations in Africa. Better mapping and enumeration of human and animal populations in rangeland environments is required to identify deficiencies in human and animal health coverage. Subsequently, the adoption of field-forward technology and surveillance in national healthcare systems can be used to extend capacity to underserved areas, where it can be deployed by One Health extension personnel.

The first step is to improve mapping and enumeration of pastoralists, their livestock, and coexisting wildlife. Accurate information on the distribution, movement patterns, and overlap of humans and animals is lacking across much of the African continent, but is required to understand the level of investment in animals and their contributions to pastoralists’ livelihoods, and predict where EIDs circulate in wildlife and livestock (e.g., Rift Valley fever and Middle East respiratory syndrome coronavirus).^6^ These data are also critical to understand how and where these pathogens might emerge into people, direct surveillance efforts, and inform delivery of human and veterinary health care.^8^ With open-source, high-cadence satellite imagery becoming increasingly accessible, advances in cloud computing and artificial intelligence enable automated interpretation of aerial data, which could be used to generate near real-time assessments of human and animal populations in remote areas. Efforts are underway to develop such a system for the enumeration of pastoralist communities in Chad^9^ and Ethiopia.^10^ Once enumerated, the information should be used to identify high-risk, underserved populations, and could facilitate emergency response efforts.

The second step is to build integrated human and animal “One Health” surveillance systems to position diagnostic testing capacity closer to the point of care, and link pastoralists, their livestock, and overlapping wildlife populations with centralized disease reporting. Integrating community-based syndromic surveillance and participatory epidemiology into existing infrastructure would promote early detection of outbreaks in humans, their livestock, and sympatric wildlife, as well as operationalizing testing and tracing capacity for future pandemics. Kenya, which has a government One Health office,^11^ has started adopting this model with the Kenya Animal Biosurveillance System using data collected by community-based animal and human surveillance officers. A similar model has been successfully deployed by the Jigjiga One Health Initiative in Somali regional state, Ethiopia. For diagnostic purposes, platforms capable of identifying occult pathogens in real time must be located closer to pastoralist communities, feeding back into local health systems to direct treatment, while simultaneously contributing to international surveillance efforts (e.g., Global Burden of Disease and Global Burden of Animal Diseases programs).^12^ Portable serology, next-generation sequencing, telemedicine, and remote Internet access tools herald a transformation in the extension of existing diagnostic technologies out of the laboratory and into the field.^13^

Finally, investment in staffing and training for frontline workers including community health officers, laboratory technicians, para-veterinarians, and wildlife rangers is required to strengthen surveillance efforts, build local capacity, and link public health laboratories to pastoralist communities. “One Health” extension personnel should be equipped with the skills and equipment necessary to conduct antemortem examinations, collect biological samples from humans and animals, and implement and interpret portable diagnostic assays, as shortcomings in these areas frequently hinder diagnostic capacity.

Failure to include mobile pastoralists and their animals in national health systems could have grave consequences. Climate change is projected to expand the risk envelope for emerging zoonotic diseases in sub-Saharan Africa’s drylands,^14^ and latent amplification of a novel pathogen outside the reach of current health surveillance infrastructure poses a major threat to global health security.

## Supplemental materials

Supplemental materials
