# Supplementary material for: Africa’s Nomadic Pastoralists and Their Animals Are an Invisible Frontier in Pandemic Surveillance
Source: Am J Trop Med Hyg. 2020 Sep 10;103(5):1777–9. doi: 10.4269/ajtmh.20-1004 (PMC7646752; doi:10.4269/ajtmh.20-1004)
Supplement: Supplementary file 1 [file tpmd201004.SD1.pdf]

Supplementary Information:

Database searches

1. ((covid[Title/Abstract]) OR (coronavirus[Title/Abstract]) OR (sars-cov-2[Title/Abstract]) OR (pandemic[Title/Abstract])) AND (africa[Title/Abstract]) NOT (mers[Title/Abstract]) date restriction 2020 – yields 187 results

2. ((covid[Title/Abstract]) OR (coronavirus[Title/Abstract]) OR (sars-cov-2[Title/Abstract]) OR (pandemic[Title/Abstract])) AND (africa[Title/Abstract]) AND (nomad* OR pastoralis* OR herd*) NOT (mers[Title/Abstract]) – yields zero results
